# Foreign

**Published:** 1841-05-22

**Authors:** 


### FOREIGN.

*Lecture on Stone in the Bladder, and Lithotomy.* By Bransby B. Cooper, Esq., F. R. S.—Gentlemen: As there is a man now in the house on whom I shall perform the operation of lithotomy in the course of a few days, I shall direct your attention this morning to the disease for the relief of which that operation is performed, and then demonstrate the successive steps of the operation on the subject you see before you. I do so in order that you may have the opportunity of comparing the account I give you of the symptoms and progress of the disease, with that which you may obtain at the bedside of this patient; and, secondly, that you may thoroughly understand what you will see in the operating theatre, and be enabled to judge if my precept and example vary.

The disease, then, is stone in the bladder, and the cause of the disease is commonly dependent on derangement of the organs of digestion and assimilation. We have lately gone over these organs in the anatomical lectures, and you will therefore readily understand how the integrity of the blood depends upon the perfect manner in which they perform their functions; and it then naturally follows, as all the secretions are derived from the blood, that if any of the usual constituents of this fluid be in excess, or if it contain substances which it does not in the healthy state, the secretions will also be altered in their physical condition and chemical composition. Thus, under certain states of the digestive organs, free acid is secreted by the kidneys, which decomposes the lithate of ammonia always found in healthy urine, uniting with the ammonia, and setting the insoluble crystalline lithic acid free, forming the well known lateritious sediment. Sometimes the lithic acid is formed in such quantity that it is deposited in the vessel soon after the urine is voided, but more frequently it does not separate till the urine has cooled, it being soluble in moderate quantity in the warm urine. In this case no mischief may result, but in the former it is as liable to separate in the urinary passages, as in the earthern vessel, and then you have a nucleus formed, which, by the incrustation of successive deposits, is converted into a urinary calculus. You should always attend to the state of the urine in cases where there are any symptoms of derangement of the urinary organs, because, as you may readily understand, you may so modify it as to prevent the occurrence of deposition.

Now with regard to the symptoms denoting the formation of a calculus: it is very remarkable that in some cases there is no pain whatever, and sometimes the first notice the surgeon has of the case, is being called upon to relieve symptoms produced merely by the mechanical obstruction of the stone to the passage of the urine. I was reading the other day in Pepy's Diary, an account of an Alderman Adams, who had made no complaint, and had not suffered from a single symptom of urinary disease, till retention of urine came on, and he died; when, on examination of his body, a stone was found in his bladder which weighed twenty-five ounces. I was once called to a man, a patient of Mr. Harrison, of Hodsden, who had never been affected with the symptoms of stone, but was suffering from retention of urine. I passed a catheter, and found an obstruction in the membranous portion of the urethra, evidently caused by the presence of a calculus, which I removed by an incision through the perineum, but, on passing the instrument on into the bladder, we found that this cavity was nearly filled by a very large stone. The man afterwards came into this hospital, and died with his constitution completely broken up. After death we removed a stone from the bladder of immense size. However, these are exceptional cases, for, in most instances, where urinary concretions have formed, their presence is soon denoted by a well-marked train of symptoms. Unless some foreign body has found its way into the bladder, and forms a nucleus, the kidney is the first seat of the morbid deposit, and then you have pain in the loins, sickness, numbness of the thigh, and retraction of the testicle. If the concretion does not pass on, inflammation is set up in the kidney, and you have acute inflammatory fever, with pain in the loins, and intolerance of pressure. In some cases abscess forms, which points in the loins, and, bursting, the stone finds an exit; and there are cases on record where stones have been removed from the kidney by surgical operation.

It is much more common, however, for the stone to pass from the kidney into the ureter, and then the symptoms I have just enumerated become greatly aggravated. You have violent pains in the groin and loins, with spasmodic retractions of the testicle, nausea, and vomiting, with faintings, sometimes approaching to a state of collapse. The stone either passes on into the bladder, or stops in the ureter. In the latter case, inflammation is excited, adhesions of the peritoneum unite the ureter to the loins, the calculus ulcerates through the ureter, suppuration is set up in the soft parts, and the stone is discharged. I once saw a case of this kind with Sir Astley Cooper. He took me over to Limehouse, to see a gentleman who had an abscess, which was supposed to depend on disease of the hip joint; and, on opening this abscess, he put his finger to the bottom of the wound, and, feeling something hard and rough, thought it was a piece of exfoliating bone, but, when he had removed it by the forceps, it proved to be a urinary calculus. Well, after this, by a patient investigation of the symptoms, he was enabled to trace their cause from its formation in the kidney to its passage along the ureter, and then, as it had not reached the bladder, it had evidently passed through the ureter by ulceration, and excited suppuration in the gluteal region. A somewhat similar case to this fell under the notice of Boyer, a French surgeon. His patient had suffered from a train of symptoms clearly referrible to the passage of a calculus along the ureter, but they were not followed by the relief commonly experienced when the stone passes into the bladder. The suffering continued, the pain in the loins was acute, and much increased after any quantity of fluid had been taken. On sounding, Boyer could detect no stone in the bladder, but, on passing his finger into the rectum, he found a tumour behind the prostate, and discovered that a stone was impacted in the ureter, just where the latter enters the bladder. He therefore performed what is called the recto-vesical operation, cutting into the bladder through the rectum, and then withdrew the stone with a pair of forceps. This I believe to be a unique case, at least where the operation was successful. You may, then, have to operate for the removal of a stone when it is detained in the ureter, but, generally, all that is required of you is to adopt means to lessen the suffering of the patient during its passage into the bladder, and, by counteracting spasm, to facilitate its transit. For this purpose general bleeding is sometimes, though not very often, necessary; cupping on the loins, with a full opiate, a warm bath, and a cathartic enema being generally sufficient for your purpose.

When the stone has entered the bladder, the symptoms in the early stage, and, indeed, in all stages, vary very consideraby in their severity; and this, not from any apparent connexion with the physical or chemical properties of the stone, for we often see very little suffering caused by the oxalate of lime, or mulberry calculus, the surface of which is very uneven, while the most excruciating agony often accompanies the phosphate of lime, a much less weighty and far smoother stone. It also often happens that where there has been a good deal of pain when the stone is small, it becomes considerably diminished when the stone has become very large; and this may probably be accounted for, from the circumstance that when the stone is very large, the urine dribbles off from the bladder, over the surface of the stone, into the urethra, without their being any contractions of the bladder; while, when the stone was small, the bladder contracted strongly upon it, and caused acute suffering. Of all the varieties of calculi, that which is called the triple phosphate, being formed of phosphate of ammonia and magnesia, is accompanied by the most suffering; and if from the presence of the phosphates in the urine, you can decide that a stone is of this nature, your prognosis will always be more unfavourable. It may be questioned, perhaps, whether there is some peculiarity of constitution which renders a patient acutely sensitive to the pain produced by a calculus, and determines the formation of the triple phosphate, or whether this stone, once have ing been formed necessarily injures the general health of the party in so marked a manner. However this may be, making allowance for the variation in the symptoms depending on the composition and size of the stone, and the constitution of the patient, soon after it has entered the bladder this organ becomes irritable, there is a frequent desire to pass water, and uneasiness after it has passed. The patients never complain of pain during the act of micturition, but afterwards; and the pain is not under the pubes in the region of the bladder, but at the under part of the glans penis, just in the situation of the frœnum. This uneasiness is relieved by bending the body forwards, and by pulling the prepuce, and thus you find the prepuce much elongated, especially in children. Then another marked and very constant symptom is, that during the flow of the urine the stream will be suddenly stopped, but will flow again on some change of position. This is evidently owing to the stone coming to the visicle orifice of the urethra, and closing the passage, and then falling away again on the position being altered. Then you always find these persons walk very cautiously; they go down stairs especially in a very careful manner, to avoid any jolting; and if by any accident they get a sudden jolt, their urine becomes bloody. It is a common question in hospitals to ask them if they came in a tax-cart, and they reply, no, for if they ever did, their urine would certainly be bloody. The urine also contains a large quantity of mucus, though it may pass clear, for mucus is soluble at 98°, the temperature of urine in the body; but when this fluid cools it deposits the mucus in considerable quantity. This is owing to a healthy action of the mucous membrane, the increased secretion being calculated to defend the delicate structure from the irritation of the stone. After a time these symptoms increase in severity, the pain becomes more violent, the desire to pass water more frequent, and the urine contains more blood and mucus. The rectum also sympathises with the bladder, and tenesmus is very constant; and it is not unfrequently the case that the patient cannot pass his water without at the same time evacuating the contents of his rectum. The amount of agony often suffered in this stage is almost beyond conception. I remember once going to Lambeth with Mr. Cline to see a patient; and this man had tied a cord across two of his bed posts, over which he threw his legs, and thus suspended himself by his hams. There was no doubt some ulceration of the bladder, and by this posture he made the stone gravitate from the ulcerated part. You may be inclined to say, when you consider what an excessively painful posture it must be to hang by hours together on a cord by the hams, that the remedy must be worse than the disease; but you must remember that pain is a comparative state; and it shows you what intense agony a man must suffer to lose the sense of pain which must follow such a position, in the relief it affords him. This man had refused an operation till it was too late,—until, indeed, it was plain the bladder was so ulcerated, that no surgeon would operate. Mr. Cline was talking over various remedies, proposing first one and then another, all of which, it appeared, had been tried, till at last he said he had seen great relief follow leak tea. Well, this was given him, and afforded the most astonishing relief, so much so, that he was enabled to resume a recumbent posture. However, it soon lost its effect, and the poor fellow died, completely worn out. I have seen it tried several times since, sometimes with and sometimes without effect; and I would advise you to bear it in mind, as worthy of trial, though you cannot foretell in what cases it will afford relief and when it will not.

The most satisfactory evidence of the presence of a stone in the bladder is obtained by sounding, that is, the passage of the instrument I hold in my hand into the bladder, when, on moving it about, you will feel it grate upon the stone; and the sound it produces by striking upon the stone is very audible. However, this means is open to mistakes; for there may be a stone in the bladder you cannot strike, and there may be a roughness of the bladder you might mistake for a stone. I am sure I have met with at least fifty cases where there were symptoms resembling those of stone in the bladder, and on passing the sound a roughness could be felt about the upper and fore part of the bladder, which a careless person might readily mistake for a stone; and, what is very singular, I never saw a single case where this roughness existed, that a stone was in the bladder. A little care will distinguish the cases, as in the former no sound is elicited by striking the rough part. In these cases I generally prescribe very strict attention to diet, with blue pill and rhubarb at bed time, and liquor potassæ, with tincture of hyoscyamus, and camphor mixture three times a day. You will, of course, regulate this by the state of the urine; but even if the urine be alkaline, I give this at first, in order to relieve the urgent symptoms, which it does, and then you can follow up the treatment by muriatic acid. I would lay it down as an axiom, that you should never perform an operation for the removal of stone from the bladder, unless you can hear the sound of the steel against the stone at the time of the operation. Do not be satisfied with the sense of touch, without that of hearing also. I distinctly felt and heard the stone in the bladder of the man now in the hospital this morning; but if I could not do so in the theatre to-morrow, I should put off the operation for that simple reason. You don't know what has occurred. The stone may have become sacculated; it may have passed out of the bladder through the urethra, or by ulceration; and in all these cases an operation would be improper.

Sometimes, on sounding, you find stone in the urethra. This may be removed either by the forceps or the knife. A child was once brought to Sir Astley Cooper, in whose urethra he distinctly felt a stone. He therefore told his man to hold the child on his knees, and separate the thighs, just as we tie them in lithotomy, and then made a cut through the perineum upon the stone. However, just as he was about to withdraw it with the forceps, it slipped back into the bladder. He determined not to leave off till he had effected his object, and accordingly carried on his incision into the bladder, and removed the stone with a pair of dressing forceps. He took his guinea, and the child was taken home in a hackney coach, and did uncommonly well; and I suppose Sir Astley is the only man who ever performed the operation of lithotomy on a mere morning patient, in his consulting room.

But let us take the common case. The stone is in the bladder, and you hear the sound strike it. I have had a stone placed in the bladder of the subject now on the table before you, and I suppose you can hear the evidence of its presence all over the theatre. The object you have in view is to remove it; and I am in the habit of dividing the operation practised for this purpose into four steps; namely, first, the opening of the perineum; second, the opening of the cavity of the pelvis; thirdly, laying open the bladder; and fourthly, removing the stone.

Now for the first step, the opening of the perineum. You see this part exposed before you, and it is evident that there must be some guide where you are to commence, and where terminate your incision. You may also see where the raphe of the perineum and scrotum become continuous with each other, and on this point I place my fore finger, and commence the first incision a finger's breadth below it, carrying it downwards and outwards, on the left side of the raphe, to a point midway between the tuberosity of the ischium and the centre of the verge of the anus. In this incision you divide the skin, superficial fascia, and the transverse muscle and artery of the perineum. You may have to stop to tie this; but I do not think I have seen more than two cases where there has been sufficient hæmorrhage to render the ligature necessary.

The perineum being thus opened, the next object is to open the cavity of the pelvis, and to do this you open the urethra just after it has passed through the deep fascia of the perineum. You bring the knife, its back towards the pubes, in contact with the groove of the staff, and then, by dividing the deep fascia in the same direction as the first incision, your second step is completed. You will sometimes see the urethra opened anterior to the point I have named,—and it is much easier to do so,—but then you would be liable to divide the artery of the bulb. I open the urethra as far back as possible; for though it causes some little delay, the safety of the patient is of more importance than any little credit gained by completing the section a few moments sooner.

The third step is to open the bladder, which is commenced by passing the point of your knife into the groove of the staff, which had already been exposed in the last step. The operator then takes hold of the staff in his left hand, and, depressing its handle, brings it parallel with the knife, which is held firmly in the horizontal position. The two instruments are simultaneously lateralized, so as to bring the cutting edge of the knife in the direction of the incisions already made. The knife is then directed onwards along the groove of the staff, through the prostate gland into the bladder, when usually an escape of urine, and the freedom of the motion of the knife, tell you that this step is completed. Your object is not to make a large incision, and you therefore keep the back of the knife in close contact with the groove of the staff; for if there is any considerable angle formed between them, of course a large incision is made as you push the knife on. It is always better to enlarge the cut through the prostate as you withdraw the knife, than to divide to any extent as it enters; but in either case you must be exceedingly cautious, for you may divide the large venous plexus of the prostate, wound the rectum or the vesiculæ seminales; or, if you divide the cellular investment of the prostate, you probably give rise to subsequent infiltration of urine into the cellular tissue of the pelvis. They say that you can tell when you have divided the prostate by the sensation given you on cutting it; but this is not the case; and if you only consider for a moment what a powerful lever you have in the long angle of this knife, you will see at once the danger you run by the slightest haste or carelessness. Enlarge your incision then as you withdraw the knife, but remember that it is only necessary to make it large enough to admit the end of your finger, as the prostate very easily tears, and the opening is enlarged far more safely by tearing than by cutting. Even if the perineum is so deep that you cannot reach the prostate with the finger, rather than run any risk with the knife, I would introduce a blunt gorget, and with this dilate the opening in the prostate. Having withdrawn the knife, you pass your finger on into the bladder, and feeling the stone, know that the third step of the operation is completed.

You have seen me perform these successive steps as I have described them, and now all the surgery of the operation is concluded; but all the real difficulty is to begin; for it is in seizing and removing the stone that the greatest mechanical dexterity is often required. You withdraw the staff, and pass the forceps into the bladder, as I now do, strike them against the stone, to discover its precise position, then gently open them, and endeavour to seize the stone. This I readily remove, drawing downwards and outwards in the direction of the external wound; but I find there is another; and now you see that, though my finger is on it, and it appears scarcely an inch from the skin of the perineum, and there are no contractions of the bladder to impede the action of the forceps, as you will meet with in operating on the living, still I have difficulty in seizing it; and now I have hold of it in the long axis. When this is the case, you must endeavour to alter its direction; but be careful not to push it out of the forceps again, as this would be very annoying, after you had been some minutes vainly endeavouring to seize it. If, notwithstanding alteration of position, you are unable to withdraw it, you must enlarge your incision.

I believe, gentlemen, that this is all I have to say to you with regard to the operation of lithotomy, with the exception of a remark or two on the straight staff, which is the one I prefer, because, by the depression of its handle, the prostate becomes so raised from the rectum as to bring the opening made through the former in a line with your external incisions, and forms, therefore, a straight passage into the bladder. You also derive another advantage from the greater space gained between the prostate and rectum, so that the intestine is much less liable to be wounded, and the removal of the stone is facilitated. With respect to the knife recommended by Mr. Key, although I generally use it myself, I certainly feel it my duty to recommend a young surgeon to use Sir Astley Cooper's probe-pointed knife for the division of the prostate gland, as the resistance which it offers, compared to the sharp-pointed instrument, gives a better indication of its having entered the cavity of the bladder, and is less liable to injure its coats.

I may also make a few remarks upon the difficulties which present themselves in the seizing and removing the stone, which may arise from the size, form, position, and consistence of the calculus, and the condition of the bladder itself—circumstances, however, of such practical importance, that I shall occupy another lecture upon the subject of lithotomy, especially with regard to the various difficulties which may embarrass the operator.—*Provincial Med. and Surg. Journ.*

---

*Biographical Sketch of Esquirol.*—John Stephen Dominic Esquirol was born at Toulouse, in 1772, of a family much esteemed in the town, as well for their private virtues as for the services which they had rendered their fellow citizens. At the beginning of the Revolution there was a frightful scarcity in France, and the mob of Toulouse was just going to call for the heads of the engrossers, when Esquirol's father, being informed that there was a stock of corn at some distance from the town, ordered it to be brought immediately, and pledged his fortune for the payment. Though he was rich, his fortune was hardly equal to so enormous an expense, and he would have been ruined, to save the lives of his fellow citizens, if the town had not discharged the debt so nobly contracted by one of its representatives. Such actions could not fail to make a lively impression on the mind of Esquirol, who, though quite young then, already possessed the high moral qualities of which his life was the continued expansion.

Esquirol began his studies at the College *de l'Esquille*, at Toulouse. He then went to the Seminary of St. Sulpice, to attend a course of philosophy; but the government having ordered this establishment to be closed, he returned to Toulouse, where he commenced his medical studies; less, perhaps, in consequence of a decided vocation for the art of healing, than with a view of obtaining the place of *officier de santé* in the army, an employment quite in harmony with the mildness of his character and the habits of his mind. After having served as surgeon in the army of the Pyrenees, he returned to Paris with the intention of continuing his medical studies. Two men, equally celebrated, and equally worthy of their celebrity, filled with ardour for science, and rather competitors for fame than professional rivals, were then lecturing on clinical medicine, and divided between them the pupils of the Parisian school: these were Corvisart and Pinel.

Corvisart, an acute observer, as well as a bold, eloquent, and enthusiastic professor, drew an immense concourse of auditors to the Charité. Pinel, hesitating and timid, not knowing how to lecture formally, but only to chat good humouredly, was followed in his visits to the Salpêtrière by a number of pupils, who, in zeal and knowledge, were nowise inferior to those who thronged the wards of the Charité, What made Pinel followed were two most valuable qualities; namely, great penetration as a practical physician, and great clearness as a professor. When you heard him discuss a disease, you would have thought that he was reading out of the book of nature. A few clear and simple propositions formed the subject of his lectures, and they were set forth in such a manner, that each hearer comprehended them without difficulty, and retained them with ease. From Corvisart one learned quickly; from Pinel, with accuracy. (*Chez Corvisart on savait vite; chez Pinel, on savait bien.*)

Esquirol attached himself to Pinel. There was such a conformity of character and tastes between the master and the scholar, that their acquaintance once formed, soon changed, on the part of Pinel, into paternal affection; on that Esquirol, into filial love.

Pinel had struck the fetters off the lunatics in the Bicêtre; he had published his immortal *Traité de la Manie*, and, with the aid of Pussin, who was placed under him as superintendent, he had effected cures, which were the more surprising, as he had to treat none but lunatics supposed to be incurable, and who had already undergone, in vain, the treatment of the Hôtel-Dieu. This was merely physical, and, with the exception of a few modifications, such as is still employed in some of our establishments. The *Nosographie Philisophique* had also appeared; and this work, which has gone through fifteen editions, had immediately become the classic book of students, and even of physicians. Whether Esquirol thought less of the *Nosographie Philosophique* than of the *Traité de la Manie* (in which case he anticipated the judgment which has since been passed on the two works,) or whether a secret inclination carried him to the study of mental diseases, he chose the latter subject, and dedicated his whole life to it. Every one knows the rough usage of lunatics before Pinel's time: they were purged, bled, loaded with chains, and confined in dungeons; while others were sent to the gallies, hung, or burned, according to the nature of their delirium, and the countries in which they lived.

In order to cure the mad, Pinel undertook to enlighten persons in their senses, and wrote his treatise on madness. The road being opened, Esquirol entered on it: he attached himself to Pinel, and followed him in his *clinique* among the lunatics at the Salpêtrière, where he collected his first cases. He admired his master's science, but was not dazzled by it. Being gifted with great penetration, a clear judgment, and wonderful activity of mind, Esquirol thought that Pinel's method might be improved, and undertook to do it. To venture to do more and better than Pinel was a great piece of boldness; yet he had this boldness, and Pinel himself applauded it.

Esquirol began by a thesis published in 1810, on "The Passions considered as Causes, Symptoms, and Curative Means of Mental Alienation." This thesis is not less remarkable for the justness of its thoughts than for the propriety and elevation of its style. Esquirol had been struck, not, as a modern says, by the analogies between madness and reason, for they are contraries, but by the points of similarity between the condition of some lunatics and that of persons agitated by strong passions; and, from the cases which he had collected on the subject, he had concluded, that madness is often a purely moral disease, and curable chiefly by moral means.

" Do you see," he said, " that man with an inflamed face, convulsed features, eyes red and sparkling? He is meditating some act of vengeance. He is uttering the sharpest and most insulting words: his voice is harsh and threatening; his phrases short, quick, and interrupted. It would seem that the organ of speech is not sufficiently moveable to express the ideas which arise in a disorderly crowd in his imagination, quickened by his wrath."

And in another place:—"Do you see that young man near the woman that he adores? His eyes are fixed; his face turns pale and red; his respiration is frequent, his words interrupted. Do not his deep sighs, and the irregular and tumultuous pulsations of his heart betray his passion? The image of her he loves pursues him; he no longer sleeps; he forgets to eat, and grows thin. Do not directly oppose his passion, for he is capable of attempting any thing to obtain the hand of the woman whom it would be in vain to refuse him. The opposition which he encounters makes his desires more energetic; he knows not the voice of his relations, and misapprehends the counsels of his friends. Wait and hope; time and absence will do what neither advice, authority, nor reason could effect."

A young Italian woman had gone mad in consequence of extreme vexation. A female friend sang some Italian airs before her, and the patient, who was much agitated and convulsed, became quiet and silent, and seemed to listen. The physician seized on this indication; he caused music to be played in the room next to the patient's and she recovered her health."

Esquoirol always recognized and proclaimed the efficacy of moral agents; and in the whole course of his long practice he employed pharmaceutical remedies only when the aberration of thought was accompanied by a material injury, characterized by some bodily symptom.

Some time after having published his thesis, Esquirol became the assistant of Pinel at the Salpêtrière; at the death of his master he succeeded him, and remained at that establishment until 1825, when he was appointed first physician at Charenton.

During his residence at the Salpêtrière, (I say his residence, because he really lived amidst his patients,) Esquirol became a writer for the great *Dictionnaire des Sciences Médicales*, in which he inserted long and really original articles on idiocy, mania, melancholy, monomania, dementia, delirium, hallucinations, and demonomania. The words *idiotie* and *monomanie* are his own, and have become classical.

To undertake to analyse Esquirol's works on madness, would be to undertake the entire history of the disease; as there is not one of the forms which it puts on that Esquirol did not observe and describe. He pointed out first to physicians, and then to magistrates, the existence of homicidal monomania, and thus saved some unfortunates from death and the infamy of the scaffold. The memoirs written by Esquirol on legal medicine are very numerous. They were chiefly on homicidal monomania and moral liberty, and were inserted in the *Annales d'Hygiène Publique et de Médecine Légale*, of which he was one of the founders and most eminent writers.

While he was chief physician to the lunatic and epileptic patients at the Salpêtrière, Esquirol gave clinical lectures on madness, which yearly attracted a great concourse of native and foreign auditors. This instruction, where the cleverness and delicate tact of the professor were mingled with his profound knowledge, formed those physicians, who subsequently, and after having distinguished themselves by their labours in this branch of the art of healing, were placed (thanks to the credit which Esquirol justly enjoyed) at the head of great lunatic establishments. In Germany, in Italy, and even in England, the majority of special physicians, those who have made madness the chief object of their studies, consider themselves fortunate in having attended the lectures at the Salpêtrière, and in being called Esquirol's pupils. They are really so, for they have read and reflected on his works, which they have translated into their language, and made the guide of their practice.

Esquirol several times visited all the lunatic asylums in France at his own expense; he described them, had plans of them engraved, and intended to publish them some day, and to set forth in detail what he has but slightly touched on, namely, the best mode of constructing and managing a lunatic asylum. This work we shall never have, as Esquirol did not leave materials sufficiently worked out, to make us certain, when we collect them together, that we have his entire scheme. Yet the asylum which was built under his direction at Ivey-sur-Seine, may make up for his silence to a certain point, for he combined there, as far as he could, all that he judged capable of contributing to the cure of lunatics. The great establishments in France destined for the treatment of lunacy, have been partly constructed in accordance with the advice and plans of Esquirol; and abroad, where his counsels and name were not less venerated than at home, we have seen a sovereign—the King of Sardinia—order a large building, originally constructed as a lunatic asylum, to be turned into barracks, and a new edifice on the plan of the French physician to be built in its stead. Esquirol formed the finest collection in existence of ancient and modern works, French and foreign, on the subject of madness; and he also collected several hundred skulls and casts of heads to assist the study of this disease. It was not for himself, but rather for the advancement of science that he had collected these valuable materials, for they were at the service of every one who wished to profit by them. To obtain this favour from him, and to be allowed to examine his MSS., it was by no means necessary to adopt his doctrines, or see as he saw. With him, every one enjoyed perfect liberty; and, if he esteemed your character, whether you were his pupil or not, obliged to him or not, you might, without fear of displeasing him, discuss a question with him, oppose your ideas to his, and, if you were in the right, he yielded with a good grace, for his self-love did not suffer in the least by it.

Though religious by nature, and a devoted partisan of the old monarchical creed, he was far from requiring any one to approve of his principles, far less to conform to his opinions. All that he asked from others was tolerance, and he every day afforded the example of this virtue. Some of his old friends spoke and thought like him; the younger ones all belonged to a party contrary to him, and yet he loved them all with the tenderness of a father. He had himself made the remark, that those in whom he had been most interested, whom he had invited to his house and admitted to his intimacy, far from sharing his opinions, had almost always attacked them. Thus he was never surrounded by flatterers, a very rare circumstance in the high position which he occupied, and which of itself would suffice for his panegyric.

Too timid to make his way in the world, as the phrase is, he owed his success to his merit alone. He neither intrigued nor caballed, he felt no jealous rivalry, nor was he in the least wretched when some competitor gained the day over him. He sometimes saw enemies attack him with animosity, and he pardoned them; he saw pupils whom he had loved turn against him, and he did not love them the less. One day when I was complaining to him of the attacks against me, and let him see how much I suffered from the whispered and printed calumnies to which I was exposed, he smiled, and consoled me by reminding me of his own history. When he published his first works he had been violently attacked; improper and abusive appellations had been addressed to him; he bore them without saying a word, and if he remembered them, it was not to accuse their authors, but to give me an example useful to my repose.

The timidity of Esquirol never degenerated into weakness, and he showed courage when duty or honour required it. At one time he defended a man suspected of aristocratic principles, and on his trial before the revolutionary tribunal at Narbonne. He spoke from his heart, his voice was warm, his look animated, and he astonished by his boldness. The audience was moved, and the relenting judges consented to be just for the nonce. At another time, when he had become a man of influence, he prevented the suppression of the college of Soreze; it was tainted with liberalism, and he was quite monarchical; but he was tolerant, and desired to convince, not to force. On another occasion he opposed the dismissal of a Montpellier professor accused of imparting revolutionary principles to his pupils, and wished the matter to be left to the cognizance of the ordinary tribunals. During the revolution of July, his house was the asylum of M. de Montbel, who was then a minister.

When Esquirol came to Paris, he was without fortune or patronage, and he lived for several years in a state approaching to distress. He liked to recall this epoch of his life to those pupils who were in a similar situation, in order to give them courage and the hope of better times.

A week before his death, and when already labouring under a pulmonary gangrene which had hindered him from seeing his patients at Charenton that day, he chose to be present at a sitting of the Council of Health, of which he was Vice President. He returned very ill, feverish, and spitting blood. He went to bed, and the pulmonary affection was of itself not serious; but he felt so weak, that from that moment, MM. Louis and Cayol, who were called in, thought his life in danger. Esquirol thought so too, and whatever trouble was taken to deceive him on this point, he prepared to die. Without having experienced any but physical sufferings, as calm in mind as when in his best health, he foresaw the day and hour of his death, prepared for it with the faith of a Christian, and expired tranquilly and without a struggle on the 12th of December, 1840, in the 68th year of his age. Esquirol was one of the most eminent physicians of our era. Henceforth his name belongs to history, and it will not perish.*

*Lond. Med. Gaz.*

---

*Bronchocele treated by Ligature.*—F. G., an exceedingly diminutive little girl, thirteen years age, was admitted October 13th, with a large bronchocele, which her friends wished to have removed, in consequence, principally, of the disfigurement which it caused. She has always had a slight swelling in the neck a little on the right side of the middle line, which has increased very rapidly within the last two years; it is now about the size of a small orange, situated in front of the sterno-mastoid, and extending from the clavicle nearly to the lower jaw; it is soft, slightly moveable, and not adherent to the skin; the pulsation of the carotid is communicated to it, when pressure is made on it backwards, but not when the tumour is grasped in the fingers placed on each side. The child does not suffer any pain in it, nor does it impede respiration, except when she laughs, and then it appears to press upon the windpipe; there is no tenderness over the tumour, and the skin is of its natural colour; the general health is pretty good; bowels regular.

Oct. 26. Mr. Liston having determined to

* Abridged from a life by M. Leuret, in the Gazette Medicale, Jan. 2, 1841.

tie the tumour, proceeded to perform the operation to-day. An incision was made over the tumour, extending through the skin, in the direction of the sterno-mastoid; the integuments were then dissected away from the subjacent growth, and held aside by assistants, so as to expose fully the base of the tumour. Mr. Liston then passed ligatures at the base of the tumour in the same manner that he employs them in erectile tumours, and fairly strangulated the diseased growth. There was pretty smart venous hæmorrhage during the operation, but this was stopped as soon as the ligatures were tied, by wrapping a strip of dry lint round the neck of the bronchocele; the patient was then put to bed; cold water to be applied over the part at short intervals.

27. The patient slept tolerably well last night. She complains this morning of some pain in the throat and head; bowels not opened since yesterday morning; tongue white; pulse frequent; still some bleeding when the patient coughs. To take a dose of castor-oil directly.

28. More comfortable; much less pain in the tumour, which is now getting darker-coloured; still slight oozing of blood occasionally.

31. Going on well; the tumour is now quite dry and black on the surface, and smells offensively; bowels kept open once a day, by giving a dose of castor oil in the morning.

Nov. 2. Hæmorrhage came on suddenly this morning at five o'clock; nearly half a pint was lost before it was arrested; the patient fainted; a piece of lint was wound firmly round the base of the tumour, and the hæmorrhage thus completely commanded. Wine and water, in small quantities, at frequent intervals, until the patient recovers.

11. Has been going on well since last report. She has been much relieved by the removal of the dead parts of the tumour; nearly the whole of it has now separated, and the wound appeared quite clean and healthy this morning, and was dressed as usual. About 1 P. M., whilst the child was eating her dinner, bleeding suddenly came on, and that so rapidly, that several ounces were lost before assistance could be obtained. It was found that the hæmorrhage was venous, and proceeded from the upper angle of the wound; it was soon checked by the application of cold, and then a graduated compress and roller were applied, and the child, who was faint and frightened, put to bed.

25. Discharged. To attend as an out-patient.

Dec. 4. Patient came to-day. Wound healed, with the exception of a very small part, which is dressed with a weak solution of sulphate of copper. There is still a little fulness and puffiness around the cicatrix. To have a draught containing iodide of iron twice a day.—*London Lancet.*

*On Congenital Opacity of the Cornea.* By Mr. S. Crompton, of Manchester.—There are two brothers in Manchester who are commonly supposed to have been born with opaque corneæ. The elder boy is eighteen years old; the younger three; and they are the second and tenth of a family of ten children of the same parents: the eyes of the rest being perfect.*

The right eyes of both brothers are staphylomatous; the staphyloma being much more prominent in the eldest boy.

Their left eyes agree in the following particulars:—They are very small, and soft to the touch; the line of union of the sclerotica and cornea is irregular, and less distinct than is natural. The irides are blue, and very convex. The eyeballs are wanting in plumpness and rotundity, and look unfinished.

An opacity of the cornea keeps a part of the pupil of the younger boy's left eye out of sight; and at the upper part, there is an irregularity in the outline of as much of it as is visible. But the elder boy has a regular pupil, and the whole of his cornea is quite transparent, saving a small portion of the lower part of it at its junction with the sclerotica, and I am not sure whether this opacity is not from an encroachment of the sclerotica at this point, and a result of the irregular line of union of the cornea and sclerotica, of which I have already spoken.

The elder boy can see well. I have endeavored to ascertain the exact degree of vision which the younger possesses, but without success. When he is examining any object, he turns his eyeball inwards and his head to one side, probably in order to present as much as possible of the pupil towards it. Things which he can handle he puts very close to his eye.

So far I have attempted to describe the *present* appearances of the eyes of these two brothers; and, in the next place, I shall lay before you the testimony upon which the supposition, that they were born blind of their right eyes, and with opaque corneæ of the left, is founded.

First, *the testimony of the mother.*—(Whom I have known seven or eight years, and can perfectly believe.) She states that on the day after he was born, she discovered that the eyes of the elder of these two boys were "not right." She was led to examine them by observing, when he was asleep, a prominence of the upper eyelid of the *right* eye. On looking at this eyeball, she discovered that it was far from being as it ought to be. It projected at that time as it does now, but not so far. The front of the *left* eye was partly covered by a

* Of the condition of the eyes of the first born child of these parents, nothing is known. The child died within a few hours of birth, and its eyes were not examined.

"pearl." This opacity grew thin first of all at the outer edge of the cornea: that portion of it, which was at the nasal margin of the cornea, being the last to gain its transparency. Her surgeon saw the eyes on the same day, and told her that medicine would be of no use to them.

Since the birth of this boy, she has always commanded her nurse to look at the eyes of her other children immediately after birth, to see if they were perfect. The third child, the fourth, the fifth, the sixth, the seventh, the eighth, and the ninth were all born with perfect eyes: the tenth was not.

Her tenth delivery was attended with danger, and her surgeon tarried awhile with her. Before he left the house, the nurse discovered that the child's eyes were not right. They were shown to him immediately, that is to say, not later than one hour after birth. He gave her the same opinion that she got respecting her eldest boy, that medicine would do no good. Most positively, there was no running of matter from this boy's eyes; there was no discharge whatever; neither were the eyes bloodshot, nor were the eyelids swollen. As to the elder boy, she cannot speak confidently. She does not remember that matter ran from his eyes; it may have done. It is so long since he was born that she is afraid to trust to her recollection.

Second, Mr. Allen, late house-surgeon to St. Bartholomew's Hospital, who attended the lady upon the birth of the youngest of these two boys, has very kindly given me the following particulars:—He saw the eyes immediately after birth, and distinctly remembers that the child was born with opaque cornea. He met Mr. Barton in consultation upon the case, and he remembers that the imperfect development of the eyeballs was particularly remarked. The child had not purulent ophthalmia.

Third, Mr. Barton, the senior surgeon to the Manchester Eye Hospital, remembers meeting Mr. Allen in consultation upon the youngest of these two children. He considered the eyeballs to be imperfectly developed,* and from the testimony, he felt assured that the opacity of the cornea was congenital.

Fourth, I saw the boy when he was about a month old. I had no doubt that the disease was congenital. There was no redness of the conjunctiva, nor granular state of the eyelids. I thought the case so remarkable that I have frequently called upon the mother to see the changes that were taking place in the eyes. At that period I made most diligent inquiries respecting the previous existence of inflammation, and satisfied myself that none had existed since birth. The entire cornea of the left eye was densely opaque. The outer edge of it cleared first of all.

Fifth, Mr. Walker, assistant surgeon to the Manchester Eye Hospital, tells me that this is the case to which he alluded in the following quotation:—

*Purulent Ophthalmia, even before birth.* By John Walker, Esq., Manchester.—"You will probably agree with me in thinking that this disease may arise from a number of causes, one of which may be some such secretion as that alluded to, and another a peculiar congenital predisposition. This extraordinary case I will state a little in detail, since, as far as I know, there is no similar one on record, although probably others must have occasionally occurred. The child, when first brought under my notice, was six months old, and the mother, a very intelligent person, informed me that at the time of birth its eyes exhibited the same appearances as were now observable. The disease had run through its entire course previously to birth, for, according to her account, there was no puriform discharge, inflammation, or intolerance of light, noticed at any time subsequently. The cornea of one eye had completely sloughed, the eyeball had sunk, and of course not the slightest vision existed.* More than one-half of the cornea of the other eye was opaque; through the remaining transparent portion a part of the pupil could be discerned, and the iris and cornea appeared almost in contact. The transparency gradually extended, and more of the pupil became accessible to light; hence, though vision was very imperfect when I last saw the child, yet it appeared to be gradually improving."—Braithwaite's *Retrospect*, vol. i. p. 115, taken from the Lancet, Feb. 8, 1840, p. 713.

Mr. Walker mentions another case which he saw on the second or third day after birth. Both cornæ "were opaque throughout, and unusually large and prominent, so that very little of the sclerotica was discernible. The opacity was of a bluish white colour; there was scarcely any irritation about either eye; nothing like

---

* Mr. Barton pointed out to me a case from Ripon, in Yorkshire, in which there was a like imperfect formation of the eyeballs. In this case the *cornea* was transparent, but the *lens* opaque. The eyes were particularly small, and it was said that the child was born with these appearances.

* I saw this child for the first time when he was about a month old; Mr. Walker when he was six. I remember distinctly that that the right eyeball was particularly small, and that none of the iris was visible in the left eye. Since that time the right eyeball has grown very considerably. It has not been a mere increase in size, but, as it were, an evolution of an eyeball. Changes are continually taking place in it. Although the cornea is staphylomatous, it appears that there is an effort to restore its transparancy. Within the last month or two, something like a pupil has become visible. It was first observed by the mother, who very anxiously watches the changes in her boy's eyes. These circumstances countenance Mr. Barton's opinion, that the eyeball had been developed, not in utero, but arrested in its growth.

inflammation." He regarded it as a case of malformation, and he says that, at the second year, the corneæ were "perfectly healthy, transparent, and of normal size."

I think it would be difficult to obtain more conclusive evidence respecting any congenital disease of the eyes than that which I have collected respecting the younger of these boys. The evidence is not so clear concerning the elder boy; but if the following cases be true, it would seem certain that several children in the same family *may* be born with opaque corneæ.

*An account of a very uncommon Blindness in the Eyes of newly-born Children.* By Mr. Samuel Farar, Surgeon at Deptford. Communicated by Mr. Watson. Read March 2d, 1790.*

"About nine years since, I was desired to see a child, who was about a month old, and apparently blind, having the cornea of both eyes opaque, so that not the least of the iris was to be seen.

"My opinion was, that nothing could be done in this case, and that the child would for ever be blind.

"About a month afterwards the parents informed me there was some alteration in the child's eyes, and requested I would examine them again. I then perceived the opacity to be so much lessened that I could faintly discern the iris. In two months more the child could perceive light, and from that period the sight progressively increased; and before it was ten months old the recovery was complete.

"About three years after, another child was born of the same parents with exactly the same appearances. Having seen the progress of the first case, I concluded that in this the event would be nearly the same, and, indeed, so it happened in much about the same space of time.

"The manner in which the cornea acquired its transparency was, in these cases, remarkably curious: the external edge, first growing thin, soon after became clear and transparent; and after this manner the whole surface of the cornea brightened up, the centre being the last spot that recovered its transparency.

"Two years ago the same persons had a third child born with the same appearances, except that the opaque part seemed thicker, and that a short round ligament, about three-eighths of an inch long, and of the thickness of a probe, arose from the inner part of the upper eyelid, was attached to the inferior edge of the cornea, and, when the eyelid lifted up, acted in some measure like an additional muscle, by partly raising the globe of the eye. The ligament soon began to waste, and in about three weeks quite vanished.

"From having seen the two preceding instances of sight restored, and from the disappearance of this ligament, I thought the opacity of the cornea in this child too would soon begin to give way; but in this I was deceived; a whole year having elapsed before the smallest alteration took place.

"At the end of a year the child seemed to be much diverted by passing its hand perpetually, with the fingers extended, before its eyes; and this has been its constant amusement from that time. The opacity has slowly diminished, but much of it yet remains.

"The child is now two years of age, but as it can find its way about the house, and distinguishes colours and different objects, by holding its head in a particular direction, I think, in time, the opacity will entirely disappear."

*Deptford*, Feb. 2d, 1790.

To recapitulate:—It is proved by the clearest testimony that in the younger of the two children whose cases I have described, the appearances existed within one hour of birth, and that there was not purulent aphthalmia. What clearer testimony can be obtained respecting any disease of the eyes being congenital?

The mother was led to examine the eyes of the elder boy on the day after birth, by observing when he was asleep a prominence of the eyelid arising from the staphylomatous cornea. The staphyloma still exists, and the mother says that the eyeball had the same appearance then, except that it was not so prominent. Her surgeon saw the eyes on the same day, and obviously considered them to be incurable by medicine.

In a third case (Mr. Walker's) the child on the second or third day after birth had perfectly opaque corneæ, and not any signs of existing inflammation.

I need not remark that it is impossible to explain the last two cases on the supposition of their having arisen from purulent ophthalmia after birth.

Instead of denoting the points of resemblance between the two first of these cases and Mr. Farar's, I must devote the remainder of this communication to an examination of the following judgment which has been passed upon the latter by Mr. Middlemore, of Birmingham:—

"The occurrence of extensive opacity of the cornea, as an effect of purulent ophthalmia of newly-born infants, appears to have led Mr. Farar into a *very curious blunder.* In a paper read before the Society for Promoting Medical Knowledge, he has very singularly pointed out, as a congenital disease, what I conceive to have been a mere effect of inflammation."* (Here Mr. Middlemore quotes Mr. Farar's papaper verbatim, as far as the end of the *second* case.)

Whether congenital opacity of the cornea

* Medical Communications, vol. 2, page 463.

* Middlemore—Treatise on Diseases of the Eye, London 1835, vol. i. p. 156.

has ever occurred, is not merely a question of curiosity, but one of practical interest. Suppose a child to be born with the appearances I have described, and that they are not observed till several days after birth, would it not be very natural for the friends to suppose it became blind whilst the doctor was seeing it daily? In defence of himself, the doctor might urge that there had been neither purulent discharge from the eyes, nor other signs of present inflammation; yet, if he turned to his own experience, and to the literature of the subject, he would find that the cases (and those, perhaps, the only cases) that he could adduce on his behalf, are disbelieved by the only author that has noticed them—by the writer of a systematic work, whose province, as such, is to pass impartial decisions, and who, being a self-elected judge, may reasonably be supposed to have regarded the cases with the greater care before he passed judgment upon them.

These remarks lead me to speak of the origin of this communication. Until I met with Mr. Middlemore's pungent remarks upon Mr. Farar, I had never doubted that Mr. Farar's cases were of the same description as those I have sent you. A very careful examination of Mr. Middlemore's criticism has led me to state the following particulars whilst they are fresh in my mind, not with the expectation of establishing that Mr. Farar's were cases of congenital opacity of the cornea, but of showing that there is nothing unreasonable in supposing them to be such, and that Mr. Middlemore's decision is hardly warranted by the facts which were before him.

The question I have to consider is, whether Mr. Middlemore, in the year 1835, was warranted, as a critic, in coming to the conclusion given above, respecting Mr. Farar's cases.

1. Mr. Middlemore states that Mr. Farar has erred in supposing the cases he describes to be congenital.

2. Mr. Middlemore conceives that the appearances resulted from purulent ophthalmia after birth.

Mr. Farar gives three cases. Of the first he says that he did not see it until it was a mouth old. He neither says that the disease was, nor that it was not, congenital. Respecting the two remaining cases, Mr. Farar positively states that he saw them, and that they were "born with" these appearances. So far of Mr. Farar's expressed opinions. Respecting the first case, I see nothing whatever to countenance Mr. Middlemore's assertion, that Mr. Farar supposed it to be congenital. But to proceed to the two remaining cases: it may be objected that, although Mr. Farar has positively said that they were congenital, there is nothing unreasonable in supposing that his belief rested upon the testimony of others. Mark the construction of Mr. Farar's narrative. Of the first case he *says* that he did not see it until a month after birth, and *does not say* that the disease was congenital. Of the second and third cases he *says* that the disease was congenital and *does not say* when he saw them. How is this "opposition" to be explained? Respecting the first case, I take it that Mr. Farar wished the reader to see that it was impossible for him to say whether the disease was congenital or not, *because* he did not see the child until it was a month old. And if Mr. Farar would not trust to the testimony of others at the end of one month, why should he rely upon it at the end of two or three days?

Again: Mr. Farar does not allude at all to the employment of medicines, or to the existence of inflammation, or of purulent discharge.

Again: Mr. Farar's cases were published in the "Medical Communications." In the preface to the first volume of that work are the following words:—"The editors of this work having formed themselves into a society for promoting medical knowledge, by collecting and publishing such papers on medical subjects as they think worthy of being preserved, now offer a volume of their collection to the public." Mr. Farar's paper, therefore, was considered by this society worthy of being preserved. Here are the names of some of the members of the society: Dr. Carmichael Smyth, Dr. S. Foart Simmons, Dr. Keir, physician to St. Thomas's Hospital, Dr. John Sims, Dr. Bland, Dr. Osborn, Dr. Douglas, Dr. Willan, Dr. Crawford, Dr. Bromfield, Dr. Gartshore, Dr. Gray, Mr. Pearson, of the Lock Hospital, Mr. Henry Watson, surgeon to Westminster Hospital (the gentleman who presented Mr. Farar's paper to the society,) Mr. Ford, of the General Dispensary, Mr. Cline, of St. Thomas's Hospital, &c. I need not say to you, Mr. Editor, that these are illustrious names: yet, illustrious though they be, if Mr. Middlemore's judgment be sound, they are fairly participators of Mr. Farar's "curious blunder." I ought not to say all of them, for that would imply that the most distinguished accoucheurs in London in the year 1790 (ten years after the publication of Mr. Ware's treatise on the purulent eyes of newly-born children) were so ignorant of purulent ophthalmia as not to have inquired at the time whether these appearances did not arise from it,—a conclusion that I cannot bring myself to admit; moreover, it would imply that, in a matter of testimony, a surgeon living fifty years afterwards is to be credited before the most distinguished physicians in London living at the very time. I have no means of ascertaining who were present at the Society when Mr. Farar's paper was read, or who decided that it was worthy of being preserved; but this is certain, that Dr. Carmichael Smyth read his well-known essay on "the danger of wounding the epigastric artery in the operation of tapping for ascites" on the same evening on which Mr. Farar's paper was read. Is it not reasonable to suppose that Dr. Carmichael Smyth would hear Mr. Farar's paper? Several persons died in London about this time from wounds of the epigastric artery in tapping, and the attention of the profession must have been generally directed to the subject; is it not very reasonable to suppose that there would be a full attendance of the members to hear a paper upon such an important subject, and written by so distinguished a man as Dr. Carmichael Smyth?

Notwithstanding the direct assertion of Mr. Farar that the two last cases were congenital; notwithstanding that it is implied in Mr. Farar's essay that he saw the children immediately after birth; notwithstanding the increased credibility of Mr. Farar's testimony, arising from the circumstances under which his paper was read to, and published by, a society consisting of the most eminent medical men in London; notwithstanding the incredible particulars which such a conclusion involves, Mr. Middlemore says that the appearances arose from purulent ophthalmia, and that Mr. Farar made a "curious blunder" in supposing them to be congenital.

Mr. Farar either is, or is not, a blunderer. I earnestly hope, for the sake of the eminent men that would be implicated, and for the honour of medicine in Great Britain after the death of the Hunters, that he is not; but that he will be found to be the first who has described an exceedingly rare form of ophthalmic disease.

The following remarks, which I have translated from one of A. Louis's admirable essays in the Memoirs of the Royal Academy of Surgery of Paris, bear so closely upon the latter part of this communication, and appear to me to be so instructive, that with your permission I will conclude it with them. They prove how dangerous it is to attempt to reason away matters of fact. Upwards of 130 years after Covillard's book was published, M. Louis has to defend him from most unjust imputations.— They are taken from "Memoire sur plusieurs Maladies du Globe de l'Œil; où l'on examine particulièrement les cas qui exigent l'extirpation de cet organe, et la méthod d'y procéder.

"Proptosis, or the complete protrusion of the eye from the orbit, presents so great facilities for amputating the globe, that it has been the general belief that there cannot be a case in which that indication is more urgent. Covillard* says that he was called to a man who had received such a violent blow on his eye with a racket ball, that the whole circumference of the eyeball was completely thrust out of the orbit. One of the wounded man's kinsmen had got a pair of scissors to sever the parts by which the eye remained attached. Our author very luckily entered just in time to stop him: and, having put back, says he, the eye into its place as judiciously and promptly as he could a cure followed. The pains he took were so successful that the wounded man was healed without any change or diminution of sight.

"Antoine Mâitre-Jan regards this case as false, but that it was founded on a fact of which the circumstances were boastingly exaggerated. He does not think that an eye completely thrust out of the orbit by a blow (although it held by some muscles or membranes,) and united again in its socket, could be contained there, grow firm, and preserve its functions. After having examined all the circumstances of the case, and refuted without consideration every thing that he could at all find fault with, he gives us those points of it which he believes to be the true ones: the ball will have struck him, says Mâitre-Jan, upon the outer canthus, where the edge of the orbit forms a sharp and prominent ridge; the conjunctiva will have been torn; this laceration, and the ecchymosis which would accompany it, would be enough to make a man who has had but little experience in these matters believe that the eye had perished, and was necessary to be taken away. Covillard interposes, and succeeds in preserving this organ. He had no obstacle to reunion to contend with, and there is no marvel, continues Mâitre-Jan, in that the vision was not at all diminished; seeing that it is possible that the eyeball had not been bruised, or if it had been bruised, it was so slightly that no part of the interior could have suffered the least derangement.

"This discussion has been transcribed in modern works, with a perfect assent to the opinions of Mâitre-Jan. The motive is highly praiseworthy. Indeed, we ought to be on our guard, and weigh the accounts and practical facts narrated by authors, before we put our entire trust in them. It behoveth that by a judicious examination of them, it be determined whether they are reasonable, and consistent with experience. But the fact given by Covillard is not the only one of the sort: we read in the observations of Lamzwerde, a physician at Cologne, of a cure like it in every respect: the injury was occasioned by a blow with a stick. The famous anatomist, Spigelius, (whom we cannot suppose capable of being deceived by appearances,) with the view of proving that the nerves are loose, and that they can be stretched very much, mentions the optic nerve as an example, in the recital of an injury done to a child by a blow with a stone, which had made the eye protrude so far out of the orbit that it hung down as low as the middle of the nose. A skilful surgeon took charge of this infant: the eye gradually reinstated itself, and so perfectly that no deformity ensued."

After mentioning the opinions of Guillemeau, Louis gives the following remarks by the anonymous editor of the fourth edition of Verduc's "Pathologie de Chirurgie," published in 1710;

* Observations Iatro-Chirurgiques, obs. xxvij. Covillard flourished about the year 1640.

"When the eyeball has been driven from the orbit by external violence, we may easily replace it therein, but we ought not to promise to be able to retain it there, much less to preserve its functions; although there have been," continues he, "authors of so bad faith as to vaunt in their observations of having done these marvellous cures, such as Joseph Covillard, amongst others, a surgeon of Montelimard, whom M. Antoine, surgeon at Merysur-Seine, has very sagaciously confuted in the tenth chapter of the excellent treatise on the eye which he has lately published: for all these narratives of impossible cures are no more regarded by true judges than the blustering of the wind."

Louis sums up by saying that "this author's scepticism and abuse will not prevail against the truth: Lamzwerde and Spigelius narrate facts which confirm that which has brought so many reproaches upon the celebrated surgeon at Montelimard."—*Lond. Med. Gaz.*

---

*Remarks on certain Muscles of the Face, and a new Muscle of the Ear.* By Professor Hyrtl, of Prague.—Numerous as are the observations of Albinus, Walther, Rosenmüller, Gantzer, Kelch, Sels, &c., on the deviations of arrangement in the muscular system; and carefully as Meckel and Soemmering have collected the more important among them in their anatomical manuals, yet certain cases occur to every practical dissector which are not contained in the works on this subject, and which therefore deserve to be made generally known. Such irregular arrangements of muscles have, in addition to the interest of novelty, a higher physiological importance, and inasmuch as they are for the most part repetitions of normal arrangements in animals, they afford the means of illustrating more clearly the relation of the organization of animals to the higher organization of man. In no part of the human body do the muscles vary so much as in the face; and the study of them is the most difficult, and consequently the most incomplete, part of the whole of myology. No dissection requires more attention, patience, and dexterity, than the demonstration of the organs of motion in the face, of their exact relations to one another, and of their manifold connections. More especially those fibres have hitherto been for the most part overlooked which go to the skin, and which, by their contractions, form the pits and the folds which regularly characterize a certain expression of the countenance.

Physiognomy might be based on more scientific foundations if anatomy were capable of pointing out what groups of muscles, and what subdivisions of them, are in action, and in what degree each acts, in embodying in the countenance any passing state of the mind, or any permanent mental constitution.

The anatomist must set himself the question, what muscles are in action in a given expression of the countenance? he should at least endeavour to explain what moving powers produce the expressions of the different states of the mind, or of the acute paroxysms of mental emotions. On these points science is still far behind-hand; for as the question now stands, all that we know is limited to the simple acts of the raising or drawing down of certain parts of the face. If in any case accuracy and a circumstantiality bordering on minuteness be necessary, it certainly is in that intricate field, in that confusion of fibres and fasciculi of muscle, which traverse the skin of the face.

The industry of Santorini, and his accuracy in the detection of the smaller fasciculi of the muscles of the face, are unequalled by any modern anatomist: on the contrary, pains have been taken to cut down his works to give them a convenient form for anatomical text-books. I have myself given much attention to the subject; I have confirmed many of the demonstrations of the excellent anatomists of former times, and have observed many things that are new. I may be allowed, therefore, to put together the observations that I have made in the following remarks.

1. The frontalis muscle arises neither from the root of the nose, or the glabella; nor, as Meckel* asserts, from the nasal process of the superior maxillary bone; but it developes its muscular fibres from an aponeurosis which covers the dorsum of the nose, and which must be regarded as the result of the interweaving of the tendons of the compressor of the nose. Hence one cannot wrinkle the forehead without at the same time moving the skin of the bridge of the nose. The layer of cellular tissue which covers the frontal muscle, and is very firmly attached to it, is, in parts, intimately connected with the subcutaneous cellular tissue (probably fascia superficialis) by means of short tough fibres, so that when the muscle contracts, and the skin covering it is thrown into wrinkles at right angles to the direction of the muscle, the depressions of the wrinkled skin correspond to the situations at which those connections exist.

The fibres of the muscle which arise from the upper border of the orbit coalesce with the corrugator supercilii, pass through the orbicularis palpebrarum,† and then turn considerably outwards on the forehead, and not unfrequently reach to the superior auris. A very constant bundle of fibres passes downwards on the dorsum of the nose, arches towards the sides, passes to the pyramidalis nasi, and then again leaves it, and loses itself in the labial portion of

---

* Handbuch der Menschl. Anat. Bd. 2 p. 480.

† This arrangement is normal and constant. Even in very weakly-muscled individuals one finds one part of the orbicularis below the other which is placed on the frontalis; the latter muscle slides as it were between the two portions of the former.

the levator labii superioris, alæque nasi. Santorini has described this fasciculus as the M. procerus.

The breadth of the frontalis muscle on each side is commonly equal to half the diameter of the orbicularis palpebrarum. In women it is generally smaller; it is broadest in heads that have a frontal suture. I have never observed a continuity of the fasciculi of the frontalis with those of the occipitalis, such as Sandifort and Ludwig say that they have seen.

2. The orbicularis palpebrarum arises not only from the ligamentum palpebræ internum and the nasal process of the superior maxillary bone, but also from the inner part of the lower border of the orbit, by tolerably numerous parallel fibres, which have hitherto been described only by Heister,* who made a musculus depressor palpebræ inferioris of them. A fasciculus also, sometimes, but not constantly, arises from the malor bone, from which also there is always sent off a small bundle of fibres, which attach themselves to the outer edge of the levator labii superioris, and remain united to it, or pass to that part of the skin in which, during laughing, the furrow forms which leads down from the side of the nose to the angle of the mouth. The fasciculus going to the zygomaticus major or minor is not always present.

It is incorrect to call the layer of muscle which extends over the tarsal cartilages a circular muscle. Accurate examination shows that the fibres of the lower and those of the upper eyelid are never continued into one another. Each lid has its own independent and separately acting fibres. This is proved not only by dissection, but by an experiment which shows that the motion of the lower eyelid is quite distinct from that of the upper. If we measure off on the border of each of the two lids an equal distance from the internal angle of one, for example the left, eye, and mark them with black dots, and then shut the eye, we can see, by looking in a glass with the right eye, that the black point on the lower lid does not correspond with that on the upper, but is carried almost a line nearer to the internal angle of the eye; which could certainly not occur if the upper and lower lids had one circular constrictor muscle.

3. The muscles of the nose can be profitably examined only in very muscular individuals. The levator labii superioris alæque nasi, which Santorini named pyramidalis, and which Winslow first described as a distinct muscle, is always connected at its origin with the constrictor of the eyelids, and sometimes with the frontalis also. We are consequently unable to close the eyelids forcibly without at the same time drawing up the alæ nasi, and wrinkling the skin on the dorsum of the nose. But before the muscle gets to the lips it sends a separate fasciculus to the skin, which is increased by a similar fasciculus from the levator labii superioris. These fasciculi together appear to perform the principal part in the formation of the wrinkles in laughing.

4. The muscle of the apex of the nose, which is figured by Santorini, is found only in very broad noses, on which I have frequently seen a small extra cartilage, lying between the triangular and alar cartilages, of an irregularly quadrilateral form, and firmly connected with the ligament which unites those two cartilages.

5. There is no depressor alæ nasi, though it is figured in all anatomical works. What Soemmering, Meckel, Hildebrandt, Krause, and others, describe as the depressor alæ nasi, is that which Santorini called the dilatator proprius pinnarum, which is in part covered by the compressor nasi (the transversalis nasi of Santorini.) If the sides of the nose be lightly touched with the fingers, and a strong inspiration be then made through the nostrils, as in taking snuff, the action of this muscle in dilating the nostrils is very distinctly felt. If I pass the tip of the little finger into one nostril, and make a similar inspiration, I feel that the short canal into which the apertures of the nostril lead acquires a more vertical direction towards the ethmoid bone, while in quiet respiration it looks more horizontally backwards towards the fauces. This is probably what Bell would call a respiratory motion of the nose; it occurs in snuff-taking, because, in snuffing up, the apertures of the nostrils are intentionally opened and shut, so that in inspiration through the nose the air passes forcibly into the nostrils, and in consequence of the more vertical position of the nasal passages carries the narcotic powder towards the upper regions of the cavities, where the terminations of the olfactory nerves ramify. In gentle inspiration, on the other hand, the more horizontally-directed passages into the nose carry the snuff towards the fauces and the larynx, as every one who breathes over finely-powdered snuff will learn by the coughing that it excites. It is only thus that we can explain why snuff and other sternutatory powders do not get into the lungs, into which they might else just as well pass as the dust of roads, which we inspire through the nostrils. In excited or difficult respiration, in severe inflammations of the lungs, and in the death-rattle, the respiratory office of this dilator of the nose is most clearly exhibited. If the nervous trunk that supplies this muscle be paralysed, the nose ceases to exercise its respiratory function. While in healthy men in deep inspiration through the nose, the nostrils are dilated by the action of this muscle, in those who are apoplectic, or whose faces are paralysed, the nostril is compressed in inspiration because the ala nasi yields to the pressure of the air like a valve, which is again raised up in expiration.

* Compend. Anat., p. 174.

6. In the skin of the cheeks I have several times, and especially in lean subjects, seen a muscular fasciculus which both originated and terminated in the skin; arising in the region of the malar bone, and ending above, or on the outer side of the angle of the mouth. It never exceeded a line in breadth; and its length varied from an inch to an inch and a quarter. It constantly presented itself in the same manner, and therefore could not be regarded as a mere accident. Its absence in men with fat cheeks might perhaps be explained by its becoming atrophied in consequence of the compression which it suffers from the accumulating adipose substance.

7. More rarely a similar fasciculus passes from the anterior edge of the tendon of the masseter to the skin of the cheek.

8. I once saw an accessory fasciculus of muscular fibres passing from the fibrous investment of the parotid gland to the zygomaticus major. The zygomaticus often gives delicate fibres to the skin, of which one, which separates from its lower edge, appears especially developed. If this accessory to the zygomaticus be pulled with the forceps, a pit is formed in the cheek at the point of its insertion in the skin.

9. The depressor anguli oris has a similar faciculus at its outer edge, which is often attached to the malar bone, and runs upwards upon it.

10. On no muscle is there so little agreement among anatomists as on the risorius Santorini. Meckel and Krause see nothing more in it than a prolongation of the platysma myoides. But this is not, and it cannot be, because the direction of the platysma crosses that of the fibres of the risorius. According to my observations it always arises from the fascia parotidea-masseterica, and never loses itself in the skin at the angle of the mouth to produce there the dimples of laughing, but is regularly connected with the insertion of the depressor anguli oris. Its discover himself regarded it as different from the platysma myoides, and said, "Alius omnino a quadrato, indeque non derivatur, quad huic ille subjicitur." It is rarely absent, and sometimes requires a breadth of two lines. It is sometimes double, and Santorini saw it even triple.

11. The platysma myoides sometimes sends a separate fasciculus over the fascia parotidea to the zygoma, where it unites with the masseter, in the same manner as a similar fasciculus from it, which passes behind the ear to the occipitalis, is attached to the outer edge of that muscle.

12. The musculus anomalus maxillæ superioris, that paradox of a muscle, has only an historical value. It proves clearly enough how easily an error in anatomy propagates itself by tradition, and how little pains have been taken to rid the science of such absurdities.

13. From the angle of the lower jaw of a man 40 years old, there arose a thin muscle two lines wide, which passed over the outer surface of the parotid to the meatus auditorius externus, to be attached to the anterior and lower border of the ridge of the meatus (musculus maxillo-auricularis.)

That this muscle has the power of enlarging the entrance of the meatus auditorius is evident from its anatomical relations. Perhaps it occurs only in men who have an acute sense of hearing. One might explain the dilatation of the external meatus when the mouth is opened (which is felt when a finger is put into the ear, and masticatory motions are performed by the lower jaw) as well by the action of this muscle as by the relation of the condyle of the lower jaw to the cartilage of the meatus.

14. In about every sixth corpse there may be found a muscle passing from the styloid process to the lower part of the circumference of the cartilage of the external meatus. It arises on the styloid process, above the origin of the stylo-glossus, with which it is connected by fleshy or tendinous fibres. It passes upwards on the outer surface of the process, gradually diminishes, and is inserted in the lowest prominence of the meatus auditorus cartilagineous by a radiating tendon. In consequence of this anotomical arrangement, this muscle acts as a depressor of the external ear, and a dilator of the meatus, and might fairly be named musculus stylo-auricularis. It is enclosed in a peculiar sheath which affixes it to the styloid process; it receives a nervous filament from the nervous occipitalis minor, and an artery from the stylo-mastoid or occipital. It is usually spindle-shaped, varies in width from half a line to a line and a half, and is sometimes two-bellied, in which case its lower portion is a fasciculus derived and passing upwards from the stylo-glossus.

I first saw this muscle when I was prosector at Vienna, in the body of a robust man, and I noted it as an interesting extra-development of muscle. In following years, and in this last, I have often found it again, and I believe I am justified in saying that it is more than a mere anomaly. In strongly muscular subjects, with short necks and tough aural cartilages, it is very rarely absent. Its influence on the dilatation of the cartilaginous meatus which leads to the membrana tympani renders it physiologically interesting. In the cases in which it is absent, there is always a tendinous band going from the origin of the stylo-glossus to the same part of the auditory passage; and this I regard as the empty sheath of the deficient muscle—a shell without its kernel.—*Ibid. from Medic. Jahrb. des. k. k. österreich. Staates.* Bd. xxx. St. 3.

